# Reduced Computed Tomography Use in the Emergency Department Evaluation of Headache Was Not Followed by Increased Death or Missed Diagnosis

**DOI:** 10.5811/westjem.2017.12.34886

**Published:** 2018-02-26

**Authors:** Daniel G. Miller, Priyanka Vakkalanka, Mark L. Moubarek, Sangil Lee, Nicholas M. Mohr

**Affiliations:** * University of Iowa, College of Medicine, Department of Emergency Medicine, Iowa City, Iowa; † University of Iowa, College of Public Health, Department of Epidemiology, Iowa City, Iowa; ‡ University of Iowa, College of Medicine, Iowa City, Iowa

## Abstract

**Introduction:**

This study investigated whether a 9.6% decrease in the use of head computed tomography (HCT) for patients presenting to the emergency department (ED) with a chief complaint of headache was followed by an increase in proportions of death or missed intracranial diagnosis during the 22.5-month period following each index ED visit.

**Methods:**

We reviewed the electronic medical records of all patients sampled during a quality improvement effort in which the aforementioned decrease in HCT use had been observed. We reviewed notes from the ED, neurology, neurosurgery, and primary care services, as well as all brain imaging results to determine if death occurred or if an intracranial condition was discovered in the 22.5 months after each index ED visit. An independent, blinded reviewer reviewed each case where an intracranial condition was diagnosed after ED discharge to determine whether the condition was reasonably likely to have been related to the index ED visit’s presentation, thereby representing a missed diagnosis.

**Results:**

Of the 582 separate index ED visits sampled, we observed a total of nine deaths and 10 missed intracranial diagnoses. There was no difference in the proportion of death (p = 0.337) or missed intracranial diagnosis (p = 0.312) observed after a 9.6% reduction in HCT use. Among patients who subsequently had visits for headache or brain imaging, we found that these patients were significantly more likely to have not had a HCT done during the index ED visit (59.2% vs. 49.6% (p = 0.031) and 37.1% vs. 26% (p = 0.006), respectively).

**Conclusion:**

Our study adds to the compelling evidence that there is opportunity to safely decrease CT imaging for ED patients. To determine the cost effectiveness of such reductions further research is needed to measure what patients and their healthcare providers do after discharge from the ED when unnecessary testing is withheld.

## INTRODUCTION

Headache is a common complaint in the emergency department (ED). 1 Use of imaging has increased since computed tomography (CT) was introduced in 1972. 2–4 In 2010, CTs were performed in 13.9% of U.S. ED visits, and 48% of these were of the head (HCT). 5 While this rise has been associated with a decline in rates of admission and transfer, 6 multiple sources have suggested that HCT use in the ED could be decreased through quality improvement efforts. 7–10

The use of HCT by emergency physicians (EP) for evaluation of headache varies widely, and 97% of EPs surveyed felt that at least some of the imaging studies ordered in EDs were medically unnecessary.^9^,^15^ The American College of Emergency Physiciansreleased its Choosing Wisely Campaign in 2013, which included avoiding HCTs in patients with minor head injury who are at low risk based on validated decision rules.^11^,^12^ During the 2015 Academy of Emergency Medicine Consensus Conference on diagnostic imaging in the emergency department, participants suggested that allowing providers to influence metrics could produce better quality metrics; they also suggested that knowledge translation for the optimization of diagnostic imaging use should be a core area warranting further research.^13^,^17^

The Centers for Medicare and Medicaid Services (CMS) proposed OP-15, “Use of Brain Computed Tomography in the Emergency Department for Atraumatic Headache,” to measure the proportion of HCTs performed on ED patients presenting with a primary complaint of headache that were supported by diagnosis codes; however, its methods were soon questioned.^11^,^16^ In 2012 while OP-15 was still under consideration, we implemented a quality improvement (QI) effort intended to improve the documentation of appropriate diagnoses in support of HCT ordering. As part of this QI effort we addressed some of the criticisms of OP-15 by expanding the indications for HCT and getting input from practicing EPs. Reviewing this QI effort in 2014 we observed that the proportions of HCT use decreased after EPs had reviewed their individual practice data. The primary objective of our present study was to determine whether the observed decrease in HCT use was associated with changes in proportions of death or missed intracranial diagnosis. Secondarily, we sought to determine whether proportions of subsequent cranial imaging or reevaluation of headache differed when compared between those who did and those who did not undergo HCT in the ED.

## METHODS

### Study Setting

This study was a before-and-after study reviewing electronic medical records (EMR) of patients sampled during a QI effort that took place at a 60,000-visit Midwestern, university-based ED between April 2012 and August 2014. We collected follow-up data by EMR review performed between June–August 2016. This study was approved by the local institutional review board under waiver of informed consent.

### Intervention

#### Quality Improvement Project

Our QI effort was structured to fulfill the practice improvement component of the American Board of Emergency Physicians’ Maintenance of Certification requirement. 14 This required collecting data on 10 visits per EP before and after an intervention. We performed two interventions in succession, so our QI effort yielded three epochs: pre-intervention (April–August 2012); post-education (December 2013–March 2014); and post-data review (April–August 2014) (Figure). At the end of each epoch, we sampled 10 visits for headache seen by each EP by searching the EMR for chief complaints of headache, and identifying the 10 most recent ED visits seen by each faculty EP.

**Population Health Research Capsule**

What do we already know about this issue?

A proportion of CTs performed to evaluate headache in ED patients show no acute intracranial pathology. Also, CT utilization rates vary significantly between emergency providers.

What was the research question?

Could we detect an increase in rates of death or missed intracranial diagnoses following a 9.6% decrease in head CT utilization?

What was the major finding of the study?

We observed no increase in rates of death or missed intracranial diagnoses in the 22.5 months following a reduction in head CT use.

How does this improve population health?

This study suggests that the use of collaborative, non-coercive means may enable emergency physicians to decrease head CT use in the ED without increasing death or missed diagnoses.

For our educational intervention we began by soliciting feedback from EPs on OP-15. Using this feedback we expanded the list of appropriate diagnoses supporting HCT (Table 1). We followed this with a series of emails and lectures explaining CMS OP-15. We also conducted group discussions during educational conferences and faculty meetings to educate EPs on selecting appropriate diagnoses to support HCT ordering and explaining the measurement process, highlighting common pitfalls. During group discussions we invited and answered questions. The explicit goal of education was to improve diagnosis documentation, rather than to decrease HCT ordering. This began in late 2012 and continued through 2013.

The data-review phase took place between January and March of 2014 when individual EPs reviewed their own HCT ordering practices based on data collected for the QI effort during the pre-intervention phase. These reviews occurred during individual faculty’s annual reviews with the department chair. In these meetings we presented each EP with his/her individual proportion of HCT ordering and proportion of appropriate diagnosis assignment. In cases where a HCT was ordered without the assignment of an appropriate supporting diagnosis (from Table 1) we reviewed the ED chart. In keeping with Schuur et al.’s findings, we found that in the majority of cases a more specific diagnosis than “headache” was clearly supported by information documented in the ED chart, but had not been assigned at the end of the ED visit.^16^ During each annual review we informed the EP of the specific cases where HCT was not supported by a diagnosis code and suggested an alternate, more-specific diagnosis or the addition of a secondary diagnosis that would have made this HCT appropriate according to CMS OP-15. This was followed by the post-data review phase when we sampled another 10 headache visits per EP. After our QI effort was completed, we were surprised to note that while there was no decrease in HCT use after the educational intervention, we observed a 9.6% reduction in HCT use after data review.

#### Data Collection and Measures

In 2016 we decided to use the dataset generated during the QI effort to investigate our study hypothesis: Was a decrease in HCT use followed by an increase in death or missed intracranial diagnosis? A pre-clinical medical student was trained as a reviewer by an emergency medicine attending who was a QI officer with experience in chart review. The trained reviewer then reviewed the EMR for all patients sampled during the QI effort. We reviewed each patient’s index ED note for the following: age at time of ED visit; gender; migraine history; known history of significant intracranial pathology; whether brain imaging was performed during the index ED visit; and findings from the brain imaging if performed. We reviewed all ED, neurology, neurosurgery, and primary care clinic notes as well as any HCT or brain magnetic resonance imaging (MRI) results occurring in the 22.5-month period following each index ED visit. This length of time was selected because it was the maximum window available from the last visit in the dataset at the time that data collection began.

Follow-up data included the following: whether a follow-up visit took place for a similar headache; diagnoses assigned at follow-up visits; date of follow-up visit; the service providing follow-up care; whether death was recorded in our EMR; whether brain imaging was performed in the follow-up period; and findings from brain imaging if performed. We distinguished between follow-up for any reason and those related to the ED visit as a marker for sample retention during the follow-up window. Data were entered into a standardized data collection spreadsheet. Prior to data collection we defined all terms in the spreadsheet in a data dictionary. No adjustment was made for trainee involvement or subsequent shift changes at the time of the index ED visit. A priori we defined potential missed diagnosis as the presence of any of the following conditions being found after the index ED visit: aneurysm involving the intracranial or cervical vessels; hydrocephalus; intracranial hypertension; stroke (ischemic or hemorrhagic); intracranial mass; subarachnoid hemorrhage; subdural hemorrhage; epidural hemorrhage; intraparenchymal hemorrhage; or dural sinus thrombosis.

To determine which subsequently-identified intracranial conditions should be counted as missed diagnoses we employed a board-certified EP (SL) to perform an independent review of all records where subsequent intracranial conditions were identified. This reviewer was blinded to the study hypothesis and had not been involved in or measured by the initial QI project. For each potential missed diagnosis, the independent reviewer reviewed the index ED visit note, follow-up visit notes and radiology reports before assigning a determination of whether the subsequently-diagnosed cranial condition could have potentially been diagnosed at the index visit. We labeled these as missed intracranial diagnoses.

#### Inclusion Criteria

Patients of all ages presenting to the ED complaining of headache who had been sampled for the initial QI effort were eligible for inclusion. Exclusion criteria included patients who arrived after inter-hospital transfer, patients admitted during their index visit, and those with a history of ventriculoperitoneal shunt. For patients with multiple ED visits, only the first visit was used as an index visit.

### Key Outcome Measures

The primary outcomes of interest were the proportion of death or missed intracranial diagnoses by epoch. Our secondary outcomes were the proportions of patients that followed up for evaluation of similar headache or those who had subsequent cranial imaging (CT or MRI).

### Data Analysis

We compared proportions of HCT performance across study epochs, and tested HCT ordering using chi-squared or Fisher’s exact tests, as appropriate. We identified descriptive statistics (proportions) for intracranial findings among those with HCT performed and those with neurological findings identified after the initial visit. We compared outcomes of death, missed diagnoses, identification of a follow-up visit for the same reason as the index ED visit, performance of cranial intervention and brain imaging by epoch and HCT performance. Differences were identified with the Kruskal-Wallis test and Wilcoxon rank-sum test. All tests were considered significant if p<0.05 using two-tailed tests, and analysis was completed using SAS 9.4 (SAS Institute, Inc., Cary, NC).

### Quality Assurance

The primary reviewer (MM) extracted data from all charts, and a second reviewer (DM) independently reviewed 30 randomly selected charts for quality assurance. Simple agreement (“yes” vs. “no”) was greater than or equal to 90% for key reported measures.

## RESULTS

We initially sampled 695 ED encounters for headache. After we excluded patients who were admitted to the hospital (19), transferred in after evaluation in another ED (19), or included in the study during a prior visit (75), we had a final sample of 582 separate, index ED encounters during the study period. Patient sex, age, migraine history, or pre-existing history of intracranial pathology did not vary across study epoch (Table 2). HCT performance at the index visit only varied by patient age and by patients with a known, pre-existing intracranial condition. Patients who received a CT during the index visit had a higher median age, and a greater proportion had no known, pre-existing intracranial condition. During our pre-education, post-education and post-data review epochs we observed CT ordering proportions of 33.3%, 36.7%, and 25.4%, respectively (p = 0.044) (Table 3).

### Primary Outcomes

After a 9.6% reduction in the frequency of HCT use we did not observe a statistically significant difference in proportions of death (p = 0.337) or missed diagnoses (p = 0.312) between study epochs. Across all epochs, we observed a total of nine deaths and 10 missed intracranial diagnoses (Table 4). No deaths had a missed intracranial diagnosis.

### Secondary Outcomes

Among patients who had a subsequent visit for evaluation of the same complaint as the index ED visit, 64% had not had a HCT during the index visit compared to 36% who did (p = 0.031). Among patients who had subsequent brain imaging after the index ED visit, 60% did not have a HCT during the index visit compared to 40% who did (p = 0.006) (Table 4).

## DISCUSSION

During our QI effort we did not observe a decrease in HCT after a year of educational interventions, but we observed a 9.6% decrease after providers reviewed their own data. This accords with the Institute of Medicine suggestion that feeding providers’ data back to them may be an important part of effectively changing physician behavior. 19 It is worth noting that during our QI effort we never explicitly instructed providers to decrease HCT ordering. This was motivated by the assumption that our doctors were already trying to do the right thing and avoid unnecessary testing, but that doctors might be capable of being more diligent in diagnosis assignment. The decrease in HCT ordering that we observed came after providers reviewed their own data. So this decrease appears to have resulted from a change that providers took upon themselves after being given the opportunity to look at objective data of their practice patterns and to reflect on what this data told them about their own practice. Happily, this would seem to support our initial assumption that doctors are generally trying to do the right thing.

Previous studies have found that CT pulmonary angiography (CTPA) for evaluation of pulmonary embolism could be safely decreased, thereby decreasing resource utilization without causing harm to patients.^20^,^21^ These studies used probabilistic decision models or looked at inpatient charges, limiting their generalizability to ED patients. The most compelling evidence supporting the safety and cost effectiveness of decreasing CTPA in ED patients had median hospital stays of 7.7 days and medical charges of $6,281.^22^ This was in contrast to the typical patient presenting to the ED with headache, where reduced testing may mean no testing. We found that a reduction in HCT use for the evaluation of ED patients with headache was not followed by increased death or missed diagnoses. However, the observations that those patients who returned for reevaluation of the same complaint and those who subsequently received brain imaging were more likely to have not had HCT during index visit calls the true impact of decreasing ED-based testing on overall resource utilization into question.

It may be the case that many patients simply feel that they need some sort of test to have had a thorough evaluation. This is supported by studies finding that ED patients who do not receive CT imaging for headache or for abdominal pain were more likely to return within 30 days.^23^,^24^ A previous study has observed up to three-fold variability in the proportion of HCT use for the evaluation of atraumatic headache in the ED.^15^ In our study we observed a convergence between EPs’ HCT-ordering proportions when we compared the pre-intervention to the post-data review phases; however, because our study was not designed or powered to investigate this, our observation is only suggestive.

## LIMITATIONS

This study has several limitations. As a retrospective chart review, we only had access to information contained in the EMR. Patients who did not follow up with us may have had death or missed diagnoses that we did not observe. In the pre-post study design, however, these factors are likely distributed across time periods, so we do not expect that this study type biased our findings. Approximately 86% of the sample had a subsequent visit within our EMR, suggesting that access to care was good and that the probability of patients seeking care outside our health system was low. Though we cannot exclude other causes of HCT reduction over time, there were no co-existing initiatives in place in the study institution to change HCT ordering practices.

Since we do not practice in a closed medical system, patients could have presented to other systems for care or could have died without presenting to our hospital. To address this issue, we limited the outcomes assessment to patients who received primary care within our university health system by excluding patients who were transferred in, improving the probability that we would capture events. Because of neurosurgical coverage in our predominantly rural state, nearly all patients in our region with significant intracranial pathology would be transferred to our institution for care; therefore, it is unlikely that such outcomes were not captured. This is supported by the observation that over 85% of patients in this study had another encounter in our health system within 22.5 months of the index visit.

The use of an outcome that did not account for the clinical conditions, comorbidities, or appropriateness of initial CT ordering limits the applicability of our findings. However, this type of metric was drafted as part of the proposed quality measure; so interpreting our CT ordering practices in this context parallels the outcomes that might be expected if this metric were more widely adopted. In this way, our study is pragmatic and reflects the limitations of case identification and administrative data use.

CMS OP-15 was found to be unreliable, in part because it relied upon administrative data.^16^ We addressed this issue by relying on chart review, the gold standard against which the aforementioned study compared OP-15. This resulted in a more reliable measure but at the cost of a highly labor-intensive technique.

## CONCLUSION

We observed no increase in death or missed-diagnosis proportions following a 9.6% reduction in HCT to evaluate ED patients presenting with headache. Patients who subsequently had a repeat visit for the same complaint or underwent HCT after ED discharge were more likely to have not had imaging performed during their index visit. Our study adds to the compelling evidence that there is room to safely decrease CT imaging for ED patients. Determining the cost effectiveness of such reductions requires further research to measure what patients and their healthcare providers do after discharge from the ED when unnecessary testing is withheld.

## Figures and Tables

**Figure:**
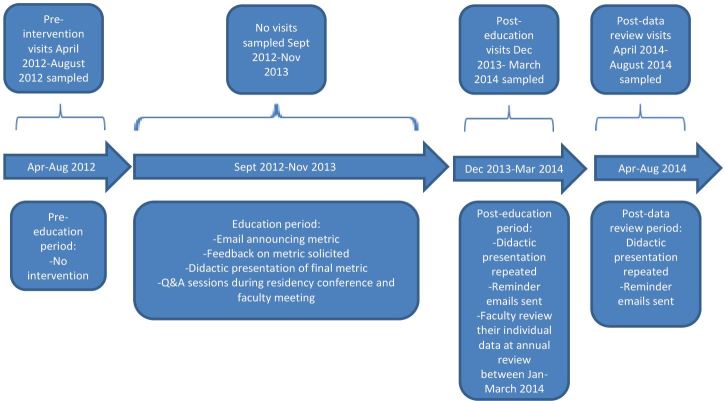
Timeline of educational phases and corresponding activities.

**Table 1:** CMS OP-15 and modifications made for QI effort.

Original CMS OP-15	Conditions added for Modified CMS OP-15
Lumbar puncture	Anticoagulant use
Dizziness	New persistent daily headache
Paresthesia	Visual disturbance
Lack of Coordination	A history of any of the following:
Subarachnoid hemorrhage	Ventriculoperitoneal shunt
Complicated headache	Neurosurgical interventions
Thunderclap headache	Coagulation or clotting disorders
Focal neurologic deficit	Subdural hemorrhage
Pregnancy	Epidural hemorrhage
Trauma	Cerebrovascular accident
HIV	Transient ischemic attack
Tumor/mass	Hydrocephalus
ED patients admitted to the hospital	Cerebral aneurysm

*CMS*, Centers for Medicare and Medicaid Services; *QI*, quality improvement

**Table 2:** Demographics and medical history of patients sampled by epoch and head CT performance.

	Overall	Epoch				CT at Index Visit		
	
		1	2	3	P value^1^	Yes	No	P value^1^
Total N(%)	582 (100.0)	174 (29.9)	215 (36.9)	193 (33.2)	***	186 (32.0)	396 (68.0)	***
Sex N(%)								
Female	367 (63.1)	113 (30.8)	1269 (35.2)	125 (34.1)	0.504	124 (33.8)	243 (66.2)	0.216
Male	215 (36.9)	61 (28.4)	86 (40.0)	68 (31.6)		62 (28.8)	153 (71.2)	
Age median (IQR)	34 (23–49)	35 (25–47)	34 (21–53)	34 (46)	0.478	43 (29–56)	31 (21–43)	<0.001
Migraine history N(%)	204 (35.1)	71 (34.8)	69 (33.8)	64 (31.4)	0.161	57 (27.9)	147 (72.1)	0.127
History of significant cranial pathology N(%)	103 (17.7)	31 (30.1)	43 (41.8)	29 (28.2)	0.421	43 (41.8)	60 (58.3)	0.019

1 Differences in categorical values determined by chi-square analysis; numerical values by Kruskal-Wallis test.

**Table 3:** Comparison of epoch and follow up by head CT ordering.

	CT at Index Visit		

	Yes; n (%)	No; n (%)	P value
Epoch			
Pre-intervention	58 (33.3)	116 (66.7)	
Post intervention 1	79 (36.7)	136 (63.3)	0.044
Post intervention 2	49 (25.4)	144 (74.6)	
Follow up			
No visit	78 (28.2)	199 (71.8)	
ED Visit	25 (28.4)	63 (71.6)	0.043
Appt-Based Visit	83 (38.3)	134 (61.8)	

*CT*, computed tomography; *ED*, emergency department.

**Table 4:** Medical outcomes and follow up by epoch and head CT order status at index visit

		Epoch				CT at index		
			
	Overall	Pre-intervention	Post-education	Post-data review	P value^1^	Yes	No	P value^1^
Outcomes	(n=582)	(n =174)	(n=215)	(n=193)		(n=186)	(n=396)	
Death	9 (1.5)	3 (1.7)	5 (2.3)	1 (0.5)	0.337	5 (2.7)	4 (44.4)	0.153
Missed diagnosis	10 (1.7)	2 (1.2)	6 (2.8)	2 (1.0)	0.312	3 (1.6)	7 (70.0)	0.893
Follow up visit for ED complaint	305 (52.7)	93 (53.5)	110 (51.2)	102 (52.9)	0.894	109 (59.2)	196 (64.3)	0.031
Cranial intervention after ED visit	21 (3.6)	8 (4.6)	8 (3.7)	5 (2.6)	0.585	9 (4.8)	12 (57.1)	0.275
Brain imaging done after ED visit	172 (29.6)	56 (32.2)	68 (31.6)	48 (24.9)	0.217	69 (37.1)	103 (59.9)	0.006
Time to follow up [Median (IQR)]^3^								
ED visit	24 (6–149)	35 (12–99)	21 (6–134)	56 (3–330)	0.688	9 (3–27)	59 (8–204)	0.012
Appt-based visit	22 (6–76)	25 (7–87)	22 (7–67)	17 (4–76)	0.920	26 (9–76)	19 (5–84)	0.637

*ED*, emergency department; *CT*, computed tomography; *IQR*, interquartile range.

1 Differences in categorical values determined by chi-square analysis or Fisher’s exact test; numerical values by Kruskal-Wallis test.

2 Outcomes were not mutually exclusive. Percentages reported represent column percentages, and chi-square analysis represent differences in each outcome by epoch.

3 Among those with a follow up only.
